# Substituting chemical P fertilizer with organic manure: effects on double-rice yield, phosphorus use efficiency and balance in subtropical China

**DOI:** 10.1038/s41598-021-87851-2

**Published:** 2021-04-21

**Authors:** Yanhong Lu, Yajie Gao, Jun Nie, Yulin Liao, Qidong Zhu

**Affiliations:** 1 Soil and Fertilizer Institute of Hunan Province, Hunan Academy of Agricultural Sciences, 730 Yuanda No.2 Rd, Furong District, Changsha, 410125 China; 2 Scientific Observing and Experimental Station of Land Conservation (Hunan), Ministry of Agriculture, Changsha, 410125 China

**Keywords:** Agroecology, Agroecology

## Abstract

Organic manure is an ideal alternative fertilizer to provide phosphorus (P) but is not fully recycled in subtropical China. In order to identify if it can replace chemical P fertilizer, a 35-year field trail in a paddy soil under double-rice cropping system was conducted to assess the effects of substituting chemical P fertilizer with pig manure (NKM) on rice yield, phosphorus use efficiency (PUE) and P balance. The N, P and K input under NKM was 1.2, 0.8 and 1.2 times of the combined chemical fertilizer treatment (NPK), respectively. The NKM treatment reached the same level of grain yield with NPK after 20 years’ application, and showed significantly 4.0% decreased double-rice grain yield compared with NPK over the 35 years. The NKM treatment reduced the crop P uptake leading to decreased PUE compared with NPK. Long-term P budget showed that NKM may result in higher potential of P loss than NPK. Thus, substituting chemical P fertilizer with organic manure under this rate of nutrient input slightly sacrificed the crop yield and may increase the P loss. Considering the benefits of soil fertility, adjusting the substitution rate with a more balanced NPK input might be alternative in subtropical China.

## Introduction

Rice is the major staple food feeding over one third of the world's population^1^. As one of the largest rice producers in the world, China had 18.4% of the global paddy rice harvested area and contributed 2.8% of the paddy rice production in 2017^2^. Red soil, an important resource for rice production, is widely distributed in tropical and subtropical regions in China. The red soil field covers approximately 22% of China’s arable land^3^, however, is known for phosphorous limitation due to the high PO_4_ anion binding capacity onto Fe/Al oxides in soil particles. In order to ensure food security or achieve greater crop yield, the application rate of mineral P fertilizers has dramatically increased over the last thirty years^4^. The overuse of mineral P fertilizers that are derived from finite reserves of phosphate rock threatens the sustainability of crop production and also results in widespread water eutrophication^5^. Organic manure, which has a relatively high P content, has been considered as an ideal soil amendment to provide P for corp growth^6^. Therefore, making full use of organic manure is an important strategy to recycle the P resources and lower the application rate of mineral P fertilizer.

Application of organic manure has positive effects on enriching nutrients capacity, increasing soil organic matter, and adjusting soil pH, etc.^7–9^. Based on the interactions of organic matter with soil P, and the indirect physicochemical effects on soil properties, the manure application can increase soil P availability usually reflected by increased crop yield, crop P uptake and P use efficiency. However, It is suggested that such effects are greatly dependent on the type of organic manure, the combined application rates of inorganic fertilizer, soil conditions, crop planting systems and the climate^10–12^. In the intensive double-rice cropping system in subtropical China, Xu et al.^13^ observed a higher yield and nutrient absorption by half chemical fertilizers combined with half swine manure application relative to full chemical fertilizers application according to a 6-years study; while fully substitution of mineral P fertilizer with cattle manure resulted in a slightly higher but insignificant mean yield compared with mineral P fertilizer application based on a 25-year experiment reported by Bi et al.^14^. However, the information about long-term manure effect on crop P uptake and P use efficiency in paddy fields in Subtropical China was still limited, and the estimation of manure contribution at an equivalent level of nutrient input is also lack^15^.

Excessive inorganic P fertilizer or organic manure application over the corp removal can move into the water bodies via infiltration, leaching or runoff, leading to the eutrophication of the aquatic environment^16–18^. Organic manure can increase the solubilized P in soil by decreasing the strength of soil P adsorption on chemical soil particles^19,20^ resulting in a higher potential of P losses compared with the use of chemical P fertilizers^15,21^. Having close interactions with the water networks and are usually flooding in the field, paddy production systems provide a critical source of P loss to the environment^22–24^. The risks of P runoff losses from five rice–wheat double-cropping systems in South China have been evaluated based on conventional management practices^25^. But the effect of long-term organic manure application on the potential of P loss in the double-rice cropping system has not been reported yet. Therefore, we hypothesized that long-term organic manure addition in replace of inorganic P fertilizer could enhance P use efficiency but also increase the P loss in the double-rice paddy field.

Based on a 35-years long-term fertilization experiment that included full chemical fertilizer treatment and substitution of inorganic P fertilizer with P-rich pig manure on paddy soil in a double-rice cropping system in subtropical China, the objectives of this study were to evaluate the effects of substituting inorganic P fertilizer with organic manure on (i) rice yield, (ii) P fertilizer use efficiency, and (iii) P input–output balance.

## Results

### Grain and straw yield

**Figure 1:**
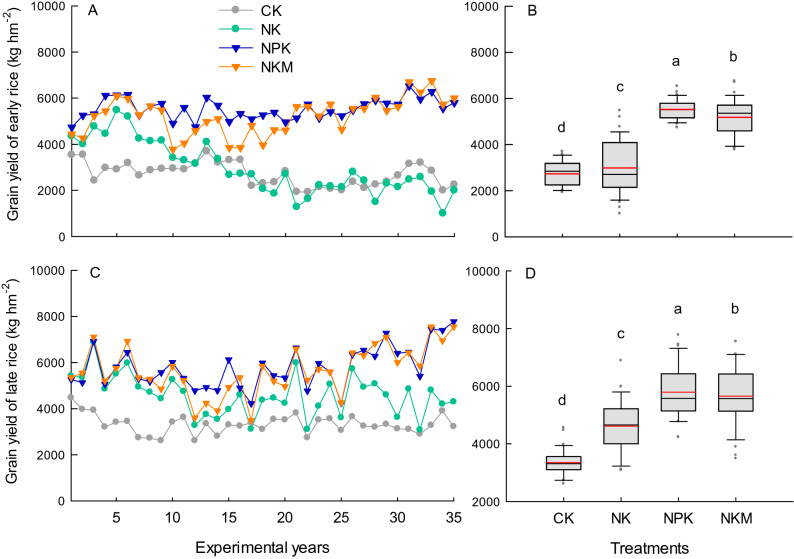
Variation and distribution of grain yields for early rice (**A**,**B**) and late rice (**C**,**D**) under different fertilization treatments CK, NK, NPK and NKM across 35 years. Red lines indicate the mean value, and different lower case letters indicate significant differences for the mean grain yield (n = 3) between treatments at p < 0.05 in (**B**,**D**).

The grain yields of early and late rice fluctuated over the 35 years and were significantly affected by the different fertilization treatments (Fig. 1). Overall, the grain yield of early rice in the NK plot declined with experimental year, while that in the CK plot was relatively stable and had less variation over the 35 years. The annual mean grain yield of both early and late rice under CK treatment (2705 and 3317 kg hm^−2^ for early and late rice, respectively) was significantly (p < 0.05) lower than that under NK treatment (2959 and 4585 kg hm^−2^, for early and late rice, respectively). The NPK treatment showed significantly (p < 0.05) higher annual mean grain yield of both the early and late rice compared with the NKM treatment. Specifically, the grain yield of early rice under NKM treatment was lower within the first 20 experimental years, while had the same level of grain yield in most of the late 15 years compared with the NPK treatment. The annual mean grain yield over 35 years under NKM treatment (5215 and 5667 kg hm^−2^ for early and late rice, respectively) was 3.6–5.7% lower (significant at p < 0.05) compared with the NPK (5533 and 5800 kg hm^−2^ for early and late rice, respectively). Moreover, the NKM treatment exhibited larger variation in grain yield of early rice, but a similar extend of variation in the grain yield of late rice relative to NPK.

**Figure 2:**
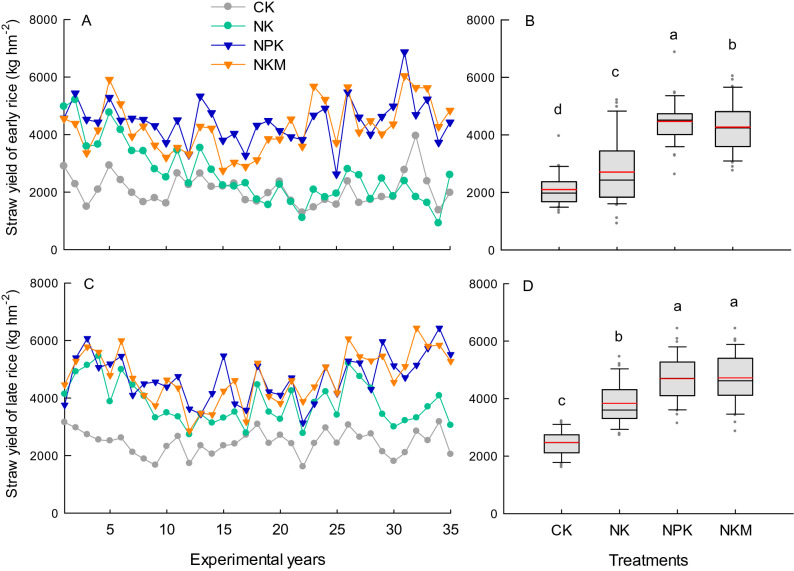
Variation and distribution of straw yields for early rice (**A**,**B**) and late rice (**C**,**D**) under different fertilization treatments CK, NK, NPK and NKM across 35 years. Red lines indicate the mean value, and different lower case letters indicate significant differences for the mean straw yield (n = 3) between treatments at p < 0.05 in (**B**,**D**).

The straw yield of both early and late rice under each treatment was 10–20% lower than their grain yield (Fig. 2). Application of either inorganic (NPK) or organic P fertilizer (NKM) increased the straw yield of both the early and late rice. The NKM treatment was 4.7% lower (significant at p < 0.05) in the annual mean straw yield of early rice, but had no significant difference in the annual mean straw yield of late rice, compared with NPK (Fig. 2). The early rice straw yield in NK plots experienced a clear decreasing trend with the experimental year, and showed the greatest variation over the 35 years among all the treatments (Fig. 2).

### P content and P uptake

**Figure 3:**
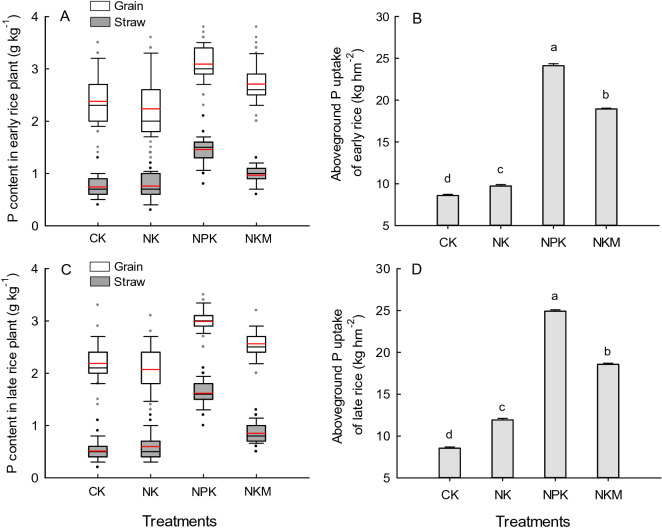
Distribution of P content in plant of early (**A**) and late rice (**C**), and the annual mean aboveground P uptake of early (**B**) and late rice (**D**) under different fertilization treatments CK, NK, NPK and NKM across 35 years. White and gray boxes indicate P content in grain and straw, respectively, and red lines indicate the mean value in (**A**,**C**). Error bars are standard error of three replicates, and different lower case letters indicate significant differences for the annual mean aboveground P uptake between treatments at p < 0.05 in (**B**,**D**).

Overall, the grain P content varied from 2.4 to 3.2 g kg^−1^ and 2.1–3.0 g kg^−1^ for early and late rice, respectively, while the straw P content varied from 0.7 to 1.5 g kg^−1^ and 0.5–1.6 g kg^−1^ for early and late rice, respectively (Fig. 3). The NPK treatment had the highest P content in either grain or straw of both early and late rice among all the treatments, showed 14% and 70% higher (significant at p < 0.05) average grain P and straw P content, respectively, compared with those under the NKM treatment. The grain P content was generally 2–3 times greater than the straw P content. Without P fertilizer application, rice plants in CK and NK plot had a higher ratio of grain P to straw P content in comparison with the P applied plots, and the NKM treatment also showed greater ratio of grain P to straw P content compared to the NPK treatment.

Application of organic or inorganic P fertilizer (NKM and NPK) significantly (p < 0.05) increased the aboveground P uptake compared with CK and NK treatment. Both the early and late rice plants in NPK plot absorbed more P (significant at p < 0.05) than those in the NKM plot, which was linked to the lower plant P content in NKM. With respect to the double-rice system, the rice crop took 49.0 kg hm^−2^ yr^−1^ P in average over 35 years under NPK treatment followed by 37.5 kg hm^−2^ yr^−1^ P under NKM treatment, and the rice crop grown in CK and NK plot took 21.7 and 17.1 kg hm^−2^ yr^−1^ P, respectively.

Analysis of variance suggested that the grain yield, straw yield, grain P content, straw P content, and aboveground P uptake were significantly (p < 0.05) affected by the fertilization, precipitation and temperature (Table 3). There was significant interaction between fertilization and temperature on the grain yield, straw yield, straw P content and aboveground P uptake in the early rice season (p < 0.01), as well as the grain yield, grain P content and the P uptake in the late rice season (p < 0.05) (Table 3). Grain P content in the early rice season, and the grain yield, straw yield together with P uptake in the late rice season were significantly (p < 0.05) affected by the interaction between precipitation and temperature (Table 3).

### PUE

**Figure 4:**
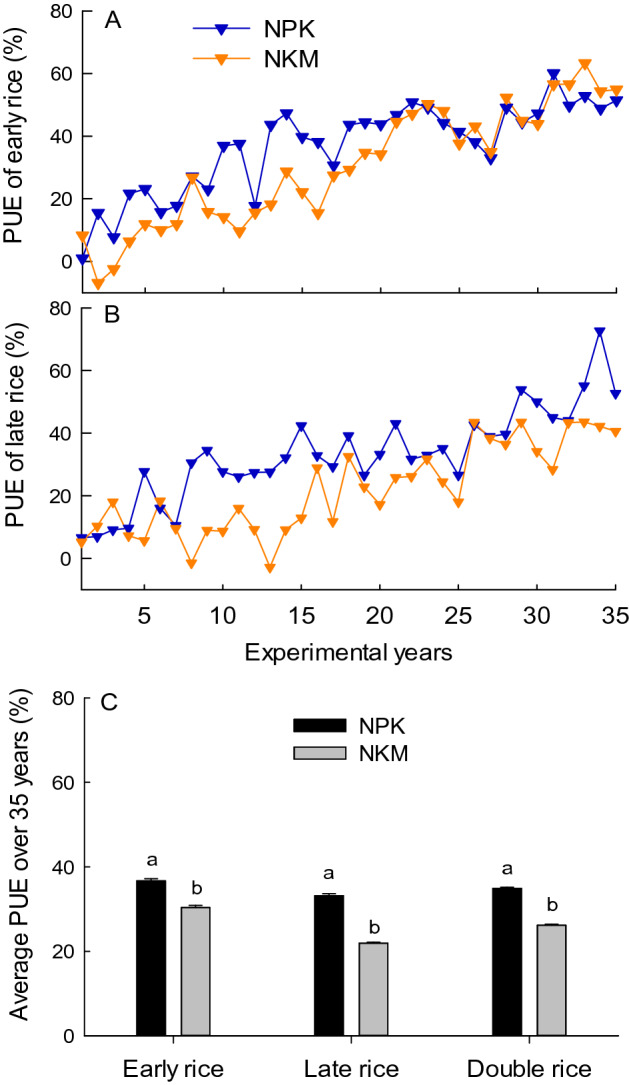
Variation of P use efficiency (PUE) for early (**A**) and late rice (**B**), and the average PUE (**C**) under NPK and NKM fertilization treatments across 35 years. Error bars are standard error of three replicates, and different lower case letters indicate significant differences between treatments at p < 0.05 in (**C**).

**Figure 5:**
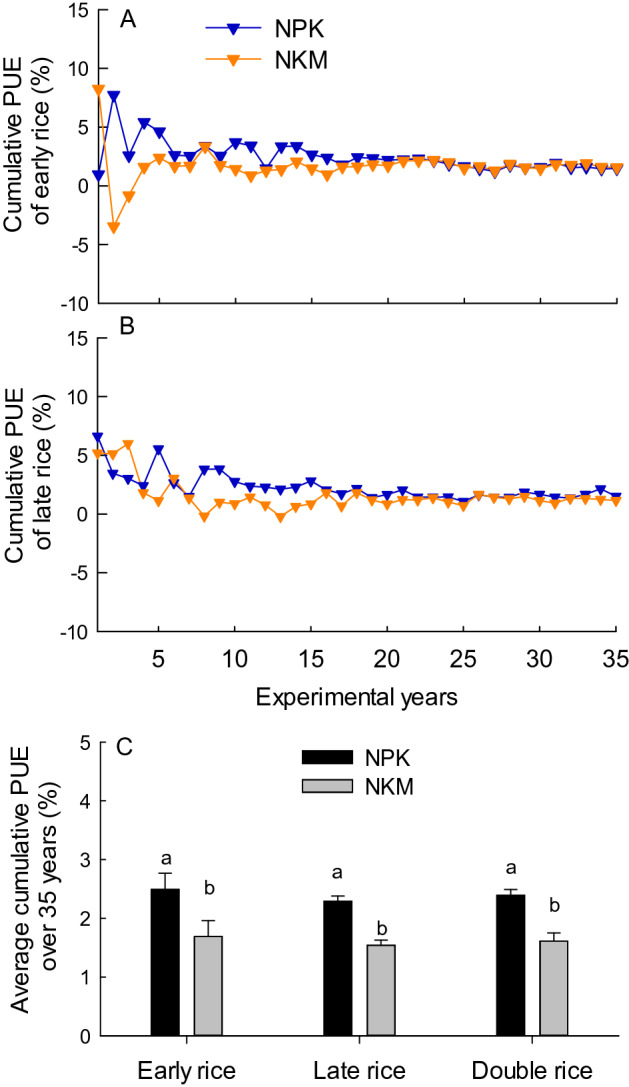
Variation of cumulative P use efficiency (PUE) for early (**A**) and late rice (**B**), and the average cumulative PUE (**C**) under NPK and NKM fertilization treatments across 35 years. Error bars are standard error of three replicates, and different lower case letters indicate significant differences between treatments at p < 0.05 in (**C**).

The application of NPK generally resulted in higher PUE for early and late rice in most of the 35 years, and was significantly higher (p < 0.05) in the average PUE over the 35 years, compared with NKM treatment (Fig. 4). The PUE of both the NPK and NKM treatment had an increasing trend with experimental year. It was due to the decreasing trend of crop P uptake under NK treatment that was subtracted by crop P uptake under NPK or NKM treatment when calculating the PUE. Therefore, to avoid the overestimation of PUE in the later period, the cumulative PUE for multiple years was further evaluated (Fig. 5). The NKM treatment had lower cumulative PUE for both the early and late rice season over the early two decades, and then in the later period had close cumulative PUE value compared with the NPK treatment. The application of NKM lead to significantly decreased (p < 0.05) average cumulative PUE over the 35 years, compared with the NPK.

### Soil P and P balance

**Figure 6:**
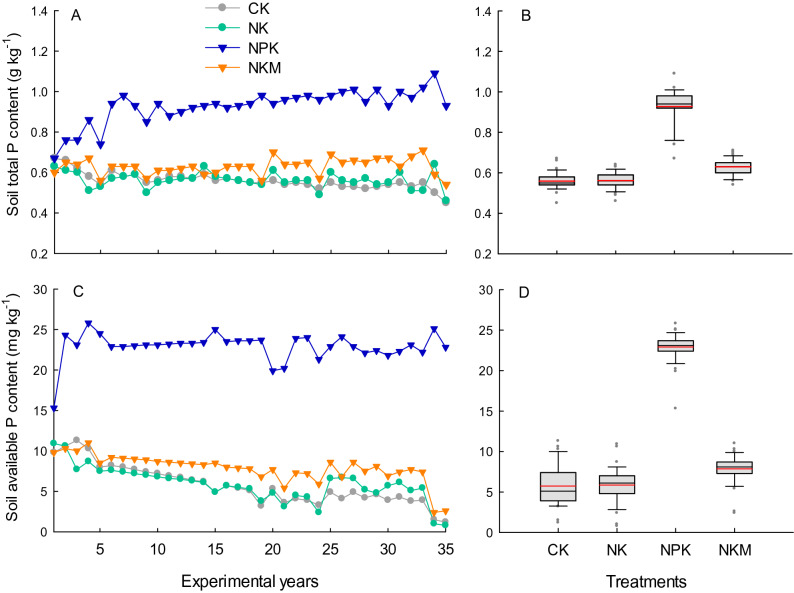
Variation and distribution of soil total P content (**A**,**B**) and soil available P content (**C**,**D**) in 0–15 cm layer under different fertilization treatments CK, NK, NPK and NKM across 35 years. Red lines in (**B**,**D**) indicate the mean value.

The soil total P and available P content in 0–15 cm soil layer over the 35 years were significantly (p < 0.05) higher under the NPK treatment, compared with those under CK, NK and NKM treatment (Fig. 6). The chemical P application (NPK) resulted in an increasing trend of soil total P content, while the NKM application had declining soil available P content with experimental years (Fig. 6, Table 1). The average total P content during 2011–2015 was 1.00 and 0.63 g kg^−1^ under NPK and NKM treatment, respectively, versus to the 0.66 g kg^−1^ in initial soil. The soil available P content increased from 10.20  to 23.09 mg kg^−1^ under NPK treatment, but decreased to 5.48 mg kg^−1^ in 2011–2015 under NKM treatment.

**Table 1:** Linear model analyzing the trend of soil total and available P content changing with experimental years (1981–2015).

Treatment	Soil total P			Soil available P		
	Slope	R^2^	*P*	Slope	R^2^	*P*
CK	− 0.003	0.624	< 0.001	− 0.220	0.843	< 0.001
NK	− 0.001	0.118	< 0.05	− 0.162	0.584	< 0.001
NPK	0.007	0.587	< 0.001	0.059	0.052	0.182
NKM	0.001	0.03	0.311	− 0.128	0.573	< 0.001

The slope is the average increase of soil total or available P content with year. R is the coefficient of the correlation between the soil total or available P content and the experimental year. CK: unfertilized control; NK: mineral fertilizer NK; NPK: mineral fertilizer NPK; NKM: mineral fertilizer NK combined with pig manure.

A complete P budget including the recorded P input, crop harvested P, and soil P storage was evaluated for the different fertilization systems over the 35 years (Table 2). Large amounts of P were removed with the harvested rice crops, showing a surplus of 11.4 and 18.1 kg P hm^−2^ yr^−1^ under the CK and NK treatment, respectively. Applications of chemical P fertilizer and pig manure exceeded the rice demand in average, showing a similar P fertilizer recovery efficiency (PRE) of approximately 62% under NKM and NPK application. Owning the lower total P storage in topsoil as a source of P output, the NKM application showed more P loss (22.1 kg hm^−2^ yr^−1^) relative to NPK application (11.8 kg hm^−2^ yr^−1^).

**Table 2:** Annual P balance over 35-year experimental period (1981–2015).

Item	CK	NK	NPK	NKM
**Input (kg hm**^**−2**^** yr**^**−1**^**)**				
Fertilizer	0.0	0.0	78.4	0.0
Pig manure	0.0	0.0	0.0	60.6
**Output (kg hm**^**−2**^** yr**^**−1**^**)**				
Corp removal	17.1	21.7	49.0	37.5
P storage in top soil layer	− 5.8	− 3.6	17.6	0.9
Calculated P loss	− 11.4	− 18.1	11.8	22.1
PRE			62.5%	61.9%

CK: unfertilized control; NK: mineral fertilizer NK; NPK: mineral fertilizer NPK; NKM: mineral fertilizer NK combined with pig manure, PRE: phosphorus recovery efficiency.

## Discussion

The current results showed that the P deficiency lead to a significant reduction in rice grain yield in the double-rice cropping system, even an adequate supply of N and K fertilizer was not able to maintain the grain yield. This result, together with the continuously declining early rice grain yield under NK treatment with experimental years indicated the P limitation for reaching a high crop yield in the paddy soil. Increasing evidences suggest that the rising temperature may threat rice production in subtropical and tropical area^26,27^. In this study, the grain yield of late but not the early rice was significantly affected by both the precipitation and temperature indicating that the late rice production can be more sensitive to meteorological instability relative to the early rice (Table 3). The significant interaction between fertilization treatment and temperature for both the early and late rice season also suggested the different responses of rice growth and plant P acquisition to temperature under different P fertilizer application. Further analysis revealed that when the grain yield under CK and NK treatment declined, the grain yield with NPK and NKM application tended to increase with rising mean temperature over rice growing season (from 23 to 24 ℃ to > 24 ℃). Those may indicate the role of P fertilization in manipulating the growth performance of rice in response to the changing temperature, and deserves to be further studied.

**Table 3:** Analysis of variance for the effects of fertilization application, precipitation, temperature and the interactions between fertilization and the other variables on grain yield, straw yield, grain P content, straw P content and aboveground P uptake.

	Grain yield	Straw yield	Grain P content	Straw P content	P uptake
**Early rice season**					
Fertilization (*F*)	***	***	***	***	***
Precipitation (*P*)	ns	**	*	*	ns
Temperature (*T*)	ns	ns	***	ns	***
*F* × *P*	ns	ns	ns	ns	ns
*F* × *T*	***	**	ns	*	**
*P* × *T*	ns	ns	**	ns	ns
*F* × *P* × *T*	ns	ns	ns	ns	ns
**Late rice season**					
Fertilization (*F*)	***	***	***	***	***
Precipitation (*P*)	*	***	ns	ns	**
Temperature (*T*)	**	***	***	***	*
*F* × *P*	ns	ns	ns	ns	ns
*F* × *T*	*	ns	*	ns	**
*P* × *T*	**	**	ns	ns	*
*F* × *P* × *T*	ns	ns	ns	*	ns

Precipitation is arbitrarily divided into three levels of < 600, 600–800 and > 800 mm for early rice season; and into three levels of < 150, 150–300 and > 300 mm for late rice season.

Temperature (monthly) is arbitrarily divided into three levels of < 23, 23–24 and > 24 ℃ for both the early and late rice season.

CK: unfertilized control; NK: mineral fertilizer NK; NPK: mineral fertilizer NPK; NKM: mineral fertilizer NK combined with pig manure, PRE: phosphorus recovery efficiency.

ns Not significant at *p* < 0.05.

* Denote significant difference in ANOVA at *p* < 0.05.

** Denote significant difference in ANOVA at *p* < 0.01.

*** Denote significant difference in ANOVA at *p* < 0.001.

The reduction of grain yield under NKM relative to the NPK application was mainly observed within the first 20 experimental years for the early rice, but not for the late rice, and the early rice grain yield under NKM treatment had greater variation relative to the NPK treatment. Those results suggested that replacement of chemical P fertilizer by pig manure with the present rate of nutrient input cannot consistently achieve a high grain yield, and the decreased yield production occurred specifically for the early rice. The different yield performance in the NPK and NKM treatment may primarily be attributed to the delaying release of nutrients into soil from organic manure. In addition, the 20% lower P input in the NKM application compared with NPK may also contributed to the decreased grain yield production under NKM treatment. However, in a 3-year experiment conducted on P deficient soils in legume-rice cropping system, even under the same amount of P application rates, Andriamananjara et al.^28^ found that dosing farmyard manure with NK but without mineral P fertilizer did not alleviate P deficiency in terms of rice yield, grain P content and aboveground P uptake. But partial (67.5%) substitution of mineral P with organic manure addition increased the rice yield compared to mineral P application alone^29^. Similar conclusions have also been proposed in other studies that chemical fertilizer cannot be fully replaced by organic fertilizer in order to achieve a high crop production^8,14,30,31^.

In the long term respect, substitution of chemical P fertilizer with pig manure (NKM) achieved the same level of rice yield and cumulative PUE under the application of NPK during 2001–2015 after 20 years’ application. The combination of organic manure with inorganic fertilizer is known to sustain or increase the crop yield benefited from the improvement of soil physic-chemical and biological properties including nutrient availability^32,33^, soil pH^34,35^, organic matter content^36^ and soil biological activity^37^. The application of pig manure in this study provided not only P but also an extra N and K fertilizer input (27 kg N hm^−2^ and 17.4 kg K hm^−2^ each rice growing season) relative to the NPK treatment, which can enhance the soil nutrient availability and benefit for the crop growth after long-term accumulation. The different application rates of N and P fertilizer between these two treatments also resulted in a 50% higher N:P supply ratio in the NKM application than in the NPK. It was reported that the proper ratio of applied N and P has a more critical role than the absolute rates of N or P supply in maximizing the crop yield, and the variations in N:P supply ratio can significantly affect the nutrient uptake^38–40^. Evidences via pot experiments suggested that the increased N:P supply ratio can significantly promote the biomass and the P uptake of graminoids^41^ and the rice plants^42^. The N and P supplied by pig manure mainly in organic form can accumulate with experimental years in this study, and the higher N:P supply ratio under long-term NKM application may therefore partially contribute to the high grain yield during the late 15 years in this study. In addition, over 35 years fertilization, the NPK application decreased soil pH from 6.60 to 4.95, while soil pH under the NKM was in most years higher than that under NPK treatment maintaining at 5.50 in average during 2011–2015 (unpublished data). Furthermore, soil under NKM application had higher organic carbon content (24.4 g kg^−1^) comparing with the NPK treatment (21.1 g kg^−1^). It has been observed that pig manure fertilization increased the yield of wheat and maize through improving organic matter content and retarding acidity in red soil from South China^8^. Hence, in the current study, substitution of chemical P fertilizer with pig manure over the 35 years is capable to sustain a high grain yield after 20 years’ application, probably benefited from the extra input of other nutrients, retarding soil acidity and the improvement of organic carbon content.

The PUE was defined as the increase of crop P uptake under P fertilization treatment relative to the no-P fertilization control produced per unit of applied P fertilizer. Substitution of chemical P fertilizer with pig manure resulted in lower PUE, which was due to the decreased crop P uptake compared with chemical P fertilizer application (Figs. 3, 4). The PUE in this study increased with the experimental years reaching to 48% and 53% in 2011–2015, and averaged at 26% and 35% over the 35 years for the double-rice system under NKM and NPK treatment, respectively (Fig. 4). The increasing trend of PUE with experimental years was directly linked to the decreasing trend of rice P uptake in no-P fertilization control, suggesting the depletion of soil residual P without P fertilizer input. In accordance with our results, a 20-year experiment in North China Plain showed increasing trend of wheat PUE from 25 to 62%^31^, whilst short-term experiment had lower PUE around 15–25%^43^, those pointed out the overestimation of PUE in the later period of long-term experiment. Therefore, the cumulative PUE for multiple years considering the cumulative effect of P fertilizer can be assistant to represent the efficiency of applied P sources utilized by crops. Within 20 years’ application, the NKM had lower cumulative PUE, while in the later stage of the long-term experiment had similar value of cumulative PUE relative to the NPK treatment (Fig. 5). This trend was tightly in agreement with the change of yield production over the 35 years (Fig. 1), which reflected the long-term positive effects of organic fertilization on the absorption rate of P by the rice crop.

The soil P levels were affected by different P fertilization treatment. Substitution of chemical P fertilizer with pig manure reduced the total P and available P content in the topsoil layer (Fig. 6). In the present study, the pig manure application provided 30.3 kg hm^−2^ P for both early and late rice, while mineral P was supplied at a slightly higher level at 39.2 kg hm^−2^ (Table 2). The less amount of P input can be one reason for the lower total P stock in soil under NKM compared to NPK application, which partially contributed to the depletion of soil available P resources under manure application (Fig. 6). In agreement with our results, Andriamananjara et al.^29^ observed that 100% substitution of mineral P fertilizer with farmyard manure resulted in significantly lower soil available P levels compared with mineral P application alone. Based on an incubation experiment on farmyard manure mineralization in tropical soils, a decreasing trend of soil resion extractable P with incubation time was also reported^44^, indicating the process of P-fixation in soil with high P absorption capacity. Previous studies show that the addition of organic materials (OM) in soil increased the P availability only supplied as the mixture of OM and mineral P compared to mineral P only at an equivalent level of P application^12,45^. In addition to the lower total P stock in soil under manure P fertilization, the adsorption of released inorganic P from pig manure onto soil surfaces possibly played a role in decreasing soil available P content, due to the strong immobilization of PO_4_ anions on the Al/Fe oxides in weathered red soil used in this study.

The manure derived inorganic P is fixed in soil or taken up by plant roots, which is suggested as a time challenging process^29^. Substitution of chemical P fertilizer with pig manure reduced the P content and P uptake of rice plants (Fig. 3), which can be attributed to the lower soil available P content under NKM compared with the NPK treatment (Fig. 6). Substitution of chemical P with manure primarily decreased the straw P content, while just lead to minor decline in grain P content compared to NPK application, such a relatively adequate P accumulation in rice grain possibly ensured the grain yield production. It is interesting that an approximately equivalent proportion (62%) of P fertilizer was recovered by crop uptake under NPK and NKM treatment over the 35 years. The results indicated that manure P application can ensure a comparable supply of P nutrient for root uptake relative to that under chemical P fertilization, although soil available P content was lower under manure P application. This is likely due to the gradual release of P from organic manure, which prevented the immediate soil P fixation, moreover, the release of organic anions during manure decomposition competed for P fixation sites on soil Al/Fe oxides leading to a reduction of soil P binding^46^, the manure derived P is thus able to be immediately taken up by corp roots.

Long-term P budget revealed that the fertilizer P input exceeded the amount of P removed by crops and stored in soil, leading to annual P loss of 11.8 and 22.1 kg hm^−2^ under NPK and NKM treatment, respectively. However, the values may have been over-estimated because the P stored in subsoil layers was not taken into calculation. Soil total P containing all the soil P pools, can be further chemically defined as the labile P, Fe-Al bound P, Ca-bound P and organic fractions, etc^47^. It is known that soil with high labile P pools has greater P availability to plants, but also higher potential for P leaching or runoff. During the 35-years fertilization, the maximum value of soil available P content after rice harvest was 25.8 and 11.0 mg kg^−1^ under NPK and NKM application, respectively (Fig. 6C), far below the predicted threshold (78 mg kg^−1^) for P leaching of the farmland in Hunan Province, China^48^. However, the concentration of soil available P can be higher just after P application than the measured value, along with the alternation of water logging and draining during the rice growing season, we supposed that the P was primarily lost via runoff with surface water. In agreement, Wang et al.^49^ reported a high potential of P runoff by P fertilizer application, which was revealed by the increased total P content in surface water, based on a 25-years study in paddy field located in subtropical China. Furthermore, the P content in surface water under combined application of inorganic fertilizer and organic manure resulted in greater potential of P runoff compared to inorganic fertilizer application alone^49^. In this study, the manure application also caused an increased P loss compared to inorganic P fertilizer application, mainly indicated by the lower soil total P storage (Table 2). This lower total P stock was likely due to the decreased P adsorption strength on soil particles via the released organic anions under manure application^19,20^. In addition, the increased soil pH^50^ and the enhanced biological reaction^51^ by manure application could also play an important role in reducing the soil P binding.

The present study investigated the long-term effects of substituting chemical P fertilizer with organic manure (NKM) on rice grain yield, P uptake, the soil P and P balance in a paddy soil in subtropical China under the double-rice cropping system. Based on the results of this 35-year field experiment, we concluded that:

1. The highest grain yield of both the early and late rice was obtained under the combined chemical P fertilizer application (NPK). Substitution of chemical P fertilizer with organic manure (NKM), which provided 1.2, 0.8 and 1.2 times of N, P and K application rates compared to NPK, showed lower early rice yield in the first 20 years, reached the similar yield level in most of the late 15 years, and decreased the double-rice grain yield by 4.0% compared with NPK over the 35 years.
2. Long-term NKM application reduced the rice P content and P uptake, leading to lower P use efficiency compared with NPK application.
3. Long-term NKM application decreased the soil total P and available P content, and had less total P storage in the topsoil layer relative to NPK application.
4. The application of NKM may result in higher potential of P loss compared to NPK based on the calculation of long-term P budget.
5. Taken together, substitution of inorganic P fertilizer with organic manure could achieve a high yield production after the long-term application, while before reaching the high yield level, it may sacrifice the grain yield of early rice. Considering the benefits of soil fertility and to maximize the rice yield and alleviate environmental pollution, partial substitution of inorganic P fertilizer with organic manure may be a profitable strategy in paddy soil in subtropical China.

## Materials and methods

### Field site and experimental designs

**Figure 7:**
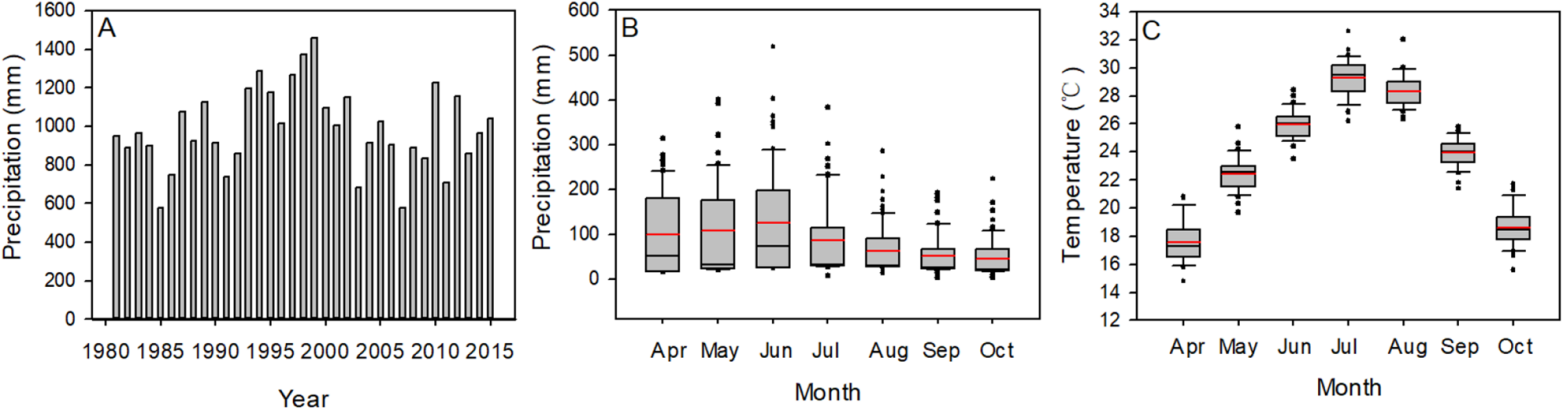
Total precipitation from April to October in each year (A), monthly precipitation (**B**), and monthly mean temperature from April to October in the years from 1981 to 2015 (**C**). Red lines indicate the mean value.

The field site of the long-term fertilization experiment was previously described by Nie et al.^52^ and Yu et al.^53^. The precipitation and monthly mean temperatures in the double-rice growing season (April to July for early rice season and August to October for late rice season) from 1981 to 2015 are shown in Fig. 7. The precipitation over double-rice growing season fluctuated during the 35 years with more than 1000 mm occurred in 16 years, 800–1000 mm occurred in 13 years, and less than 800 mm occurred in the remaining 6 years. There was no significant correlation between the precipitation and experimental years. The monthly mean temperature during the early and late rice growing season over 35 years was 23.8 °C and 23.6 °C, respectively. The monthly mean temperature also exhibited a significantly increasing trend form 1981 to 2015. The soil was a typical paddy soil derived from quaternary red clays in the subtropical China. The Characteristics of soil (0–15 cm) at the initial of the experiment in 1981 are shown in Table 4. The experiment was conducted following a double-rice (*Oryza sativa* L.) succession. The fertilization experiment was established in a completely randomized block design with three replicates. The area of each plot was 66.7 m^2^. Four treatments were evaluated: (1) CK: no fertilizer control; (2) NK: chemical fertilizer N and K application; (3) NPK: balanced chemical fertilizer N, P and K application; (4) NKM: application of chemical fertilizer NK plus pig manure. The pig manure was fermented and applied at 15 t hm^−2^ by fresh matter (85.4% water content) for both early and late rice. The nutrient contents of applied pig manure were measured every five years from 1981 to 2015, and the mean values (12.33 g N kg^−1^, 13.84 g P kg^−1^, and 7.95 g K kg^−1^ on dry weight basis) were used to calculate the amounts of N, P, K fertilizer input under NKM treatment. The early rice cultivar Zhongzao39 and late rice cultivar Shenyou9586 were used over the 35 experimental years. The planting and harvesting date, experimental designs and the type of chemical fertilizers can be seen in Yu et al.^53^. The amounts of N, P, K fertilizer application for early and late rice under different treatments were described in Table 5.

**Table 4:** Soil basal characteristics in 0–15 cm soil layer in 1981.

Characteristics		
pH		6.6
Bulk density	g cm^3^	1.12
Soil organic carbon	g kg^−1^	19.9
Total N	g kg^−1^	2.05
Total P	g kg^−1^	0.66
Total K	g kg^−1^	14.1
Alkali-hydrolyzale N	mg kg^−1^	151
Olsen-P	mg kg^−1^	10.2
Exchangeable K	mg kg^−1^	62.3

**Table 5:** Descriptions of N, P, K applications of early and late rice under different fertilization treatments.

Treatment	Early rice (kg hm^−2^)			Late rice (kg hm^−2^)		
	N	P	K	N	P	K
CK	0	0	0	0	0	0
NK	150	0	99.6	180		99.6
NPK	150	39.2	99.6	180	39.2	99.6
NKM	150 + 27	0 + 30.3	99.6 + 17.4	180 + 27	0 + 30.3	99.6 + 17.4

In the NKM treatment, the first and the second value are the amount of NPK applied as chemical fertilizer and as pig manure, respectively. CK: unfertilized control; NK: mineral fertilizer NK; NPK: mineral fertilizer NPK; NKM: mineral fertilizer NK combined with pig manure.

### Sampling and chemical analysis

Soil samples in each plot at the depth of 0 to 15 cm at five randomized points by auger boring (3.8 cm in diameter) were collected after the harvest of late rice in each year. After removing the crop residues and stone, the samples were homogenized, air dried and ground to pass through a 2-mm and a 0.25-mm sieve, respectively, labeled and then stored for further analysis. Soil pH was determined with glass electrode pH meter (soil/ water = 1:1, w/v). SOM was determined using the Walkley–Black chromic acid wet oxidation method. Total N was determined by the Kjeldahl method. Soil available P was extracted with 0.5 M NaHCO_3_ and analyzed colorimetrically according to the method described by Murphy and Riley^54^. Soil total P content was measured with the molybdovanadate method following digestion of soil in 60% HClO_4_^55^.

Each year the grain yield of both early and late rice was measured at maturity based on the entire plot area using a thresher to separate the grains from the straw. Before the entire plot harvesting, sub-samples of whole rice plants from five random hills in each plot were collected, dried at 65 °C, weighted, separated into grains and straw, and then ground for further analysis. Approximately 0.2 g of the ground plant samples was used to determine the P content following the molybdovanadate method described by Soon and Kalra^56^.

### Calculations and statistical analysis

The P use efficiency (PUE) in each growing season, the cumulative PUE for multiple years and the P recovery efficiency (PRE) were calculated based on the aboveground P uptake by rice plants and the amount of fertilizer P applied using the following equation:

$${\text{PUE }} = \, \left( {{\text{U}}_{{\text{p}}} - {\text{U}}_{0} } \right)/{\text{F}}_{{\text{p}}} \times { 1}00$$

$${\text{Cumulative PUE }} = \, \left( {{\text{U}}_{{\text{p}}} - {\text{U}}_{0} } \right)/\left( {{\text{F}}_{{\text{p}}} \times {\text{ years}}} \right) \times { 1}00$$

$${\text{PRE }} = {\text{ U}}_{{\text{p}}} /{\text{F}}_{{\text{p}}} \times { 1}00$$

where U_p_ and U_0_ represented the aboveground crop P uptake with (NPK or NKM treatment) and without P application (NK treatment), respectively, and F_p_ was the amount of P applied in the specific rice growing season.

The P balance was evaluated as the difference between P input and output. The P input was mainly considered as the applied inorganic and organic P fertilizer. The output included the aboveground crop P uptake and the residual P stored in the soil (0–15 cm layer), the latter was calculated using the following equation:

$${\text{Residual P storage }} = \, \left( {{\text{TP }} - {\text{ TP}}_{0} } \right) \, \times {\text{ D }} \times {\text{ B}}$$

where TP represented the average soil total P content in the last 5 experimental years (2011–2015) for the present soil P level. TP_0_ was the soil total P content in the initial soil. D and B represented the soil depth and soil bulk density, respectively.

Statistical analysis was carried out with Fisher’s LSD post-hoc test using the IBM Statistical Product and Service Solutions (SPSS) Statistics (Version 19.0) by taking analysis of variance (ANOVA). Differences were considered statistically significant at p < 0.05, and the significant differences between treatments were indicated by different letters.
